# 

**Published:** 1873-01

**Authors:** 


MEETING OF THE NEW YORK STATE MEDICAL SOCIETY.—The New York State Medical Society, holds its annual meeting in Albany, the first Tuesday in February, 1873. This meeting promises to be one of considerable interest, and members and delegates from County Medical Societies should not fail to be present.

### Books and Pamphlets Received.

Wöhler's Outlines of Oganic Chemistry. By Rudolph Fittig, Ph. D., Nat. Sc. D., etc. Translated from the Eighth German Edition, by Ira Remsen, M. D., Ph. D. Philadelphia: Henry C. Lea, 1873. Buffalo: T. Butler & Son,

Surgical Diseases of Infants and Children. By M. P. Guersant. Translated from the French by Richard J. Dunglison, M. D. Philadelphia: Henry G. Lea, 1873. Buffalo: T. Butler & Son.

Obstetric Aphorisms for the use of Students commencing Midwifery practice. By Joseph G. Swayne. M. D. Second American from the Fifth revised English Edition, with additions, by Edward R. Hutchins, M. D. Philadelphia: Henry C. Lea, 1873. Buffalo: T. Butler & Son.

A Handbook of Therapeutics. By Sidney Ringer, M. D. Third Edition, New York: Wm. Wood & Co., 1873. Buffalo: H. H. Otis.

Contributions to Mental Pathology. By J. Ray, M. D. Boston: Little Brown & Co., 1873. Buffalo: Jas. M. Lent.

Seventeenth Annual Report of The Trustees of the State Lunatic Hospital at Northampton. October, 1872.

Peculiarities in the Operations of the three great Ovariotomists—Wells, of London; Atlee, of Philadelphia; and Thomas Keith, of Edinburgh. By S. Fitch, M. D., Edinburgh. Philadelphia: J. B. Lippincott & Co., 1872.

THE BUFFALO

### Medical and Surgical Journal

PUBLISHED MONTHLY,

Containing Original Articles, Reports of Medical Societies and Hospitals, Editorial, Reviews, Correspondence, Army News, etc., etc.; including the usual variety of Medical Periodical Publications. Specimen copies sent on application.

Address Buffalo Medical & Surgical Journal, Buffalo, N. Y.

TERMS OF SUBSCRIPTION, - - $3.00 PER YEAR, IN ADVANCE.

